# Supplementary material for: Tumor-Associated Fibroblast-Derived Exosomal circDennd1b Promotes Pituitary Adenoma Progression by Modulating the miR-145-5p/ONECUT2 Axis and Activating the MAPK Pathway
Source: Cancers (Basel). 2023 Jun 27;15(13):3375. doi: 10.3390/cancers15133375 (PMC10340501; doi:10.3390/cancers15133375)
Supplement: Supplementary file 1 [file cancers-15-03375-s001.zip › Supplementary Figures S1-S8.pdf]

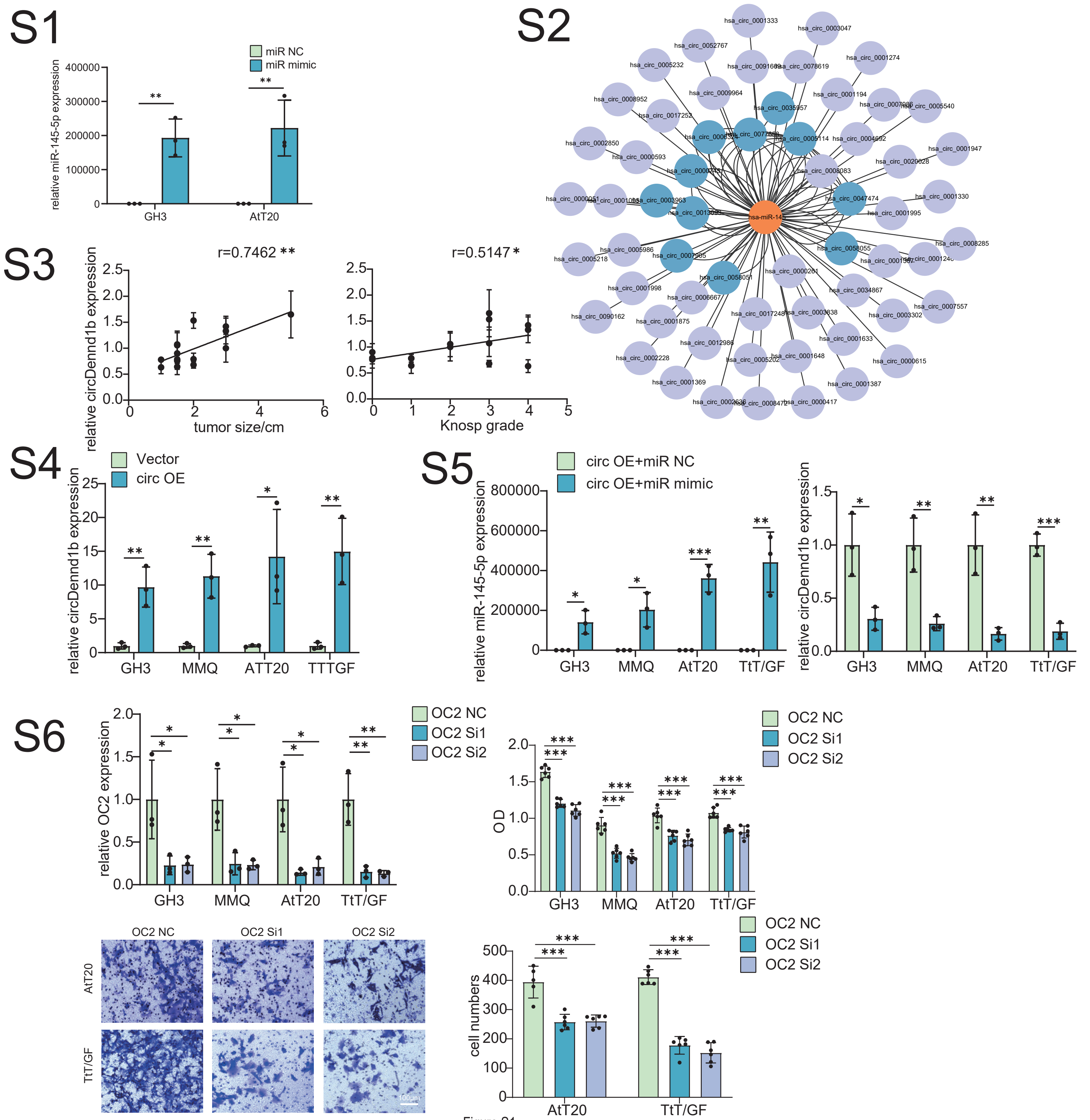

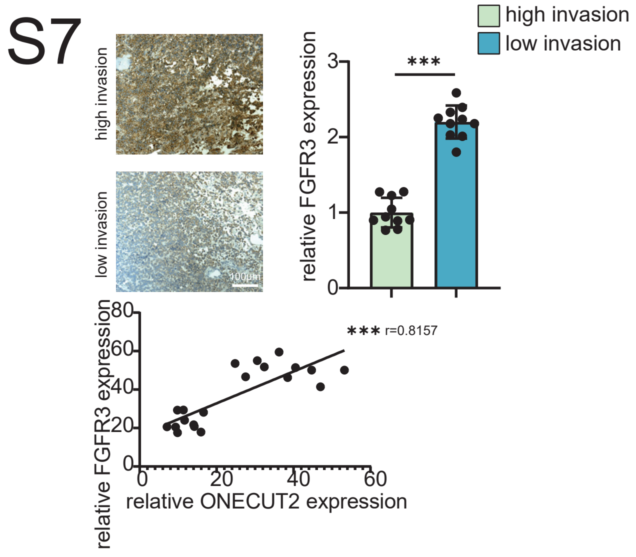

Figure S1

Verification of overexpression of miR-145-5p

Figure S2

Verification of overexpression of circDennd1b.

Figure S3

The expression level of circDennd1b correlatedwith tumor size and aggressiveness.

Figure S4

Schematic diagram of miR-145-5p binding in circDennd1b upregulated in PA.

Figure S5

Theexpression levels of circDennd1b and miR-145-5p after PA cells co-transfected with circDennd1b or Vector and miR-145-5p mimic or NC.

Figure S6

The expression level of ONECUT2 knockdown and detect the proliferation and migration abilities of PA cells after knockdown of ONECUT2 by cell viability experiments and Transwell assay.

Figure S7

FGFR3 expression was higher in pituitary adenomas in the high invasion group compared to the low invasion group, and FGFR3 expression levels were positively correlated with ONECUT2 expression levels

Pearson correlation and linear regression were used to compare these parameters between two variables. One-way ANOVA was used for multi-sample comparison and Student's t-test was used for comparison between two groups. Statistical significance was considered to be indicated by a value of $p < 0.05$.* $P<0.05$, ** $P<0.01$, *** $P<0.005$

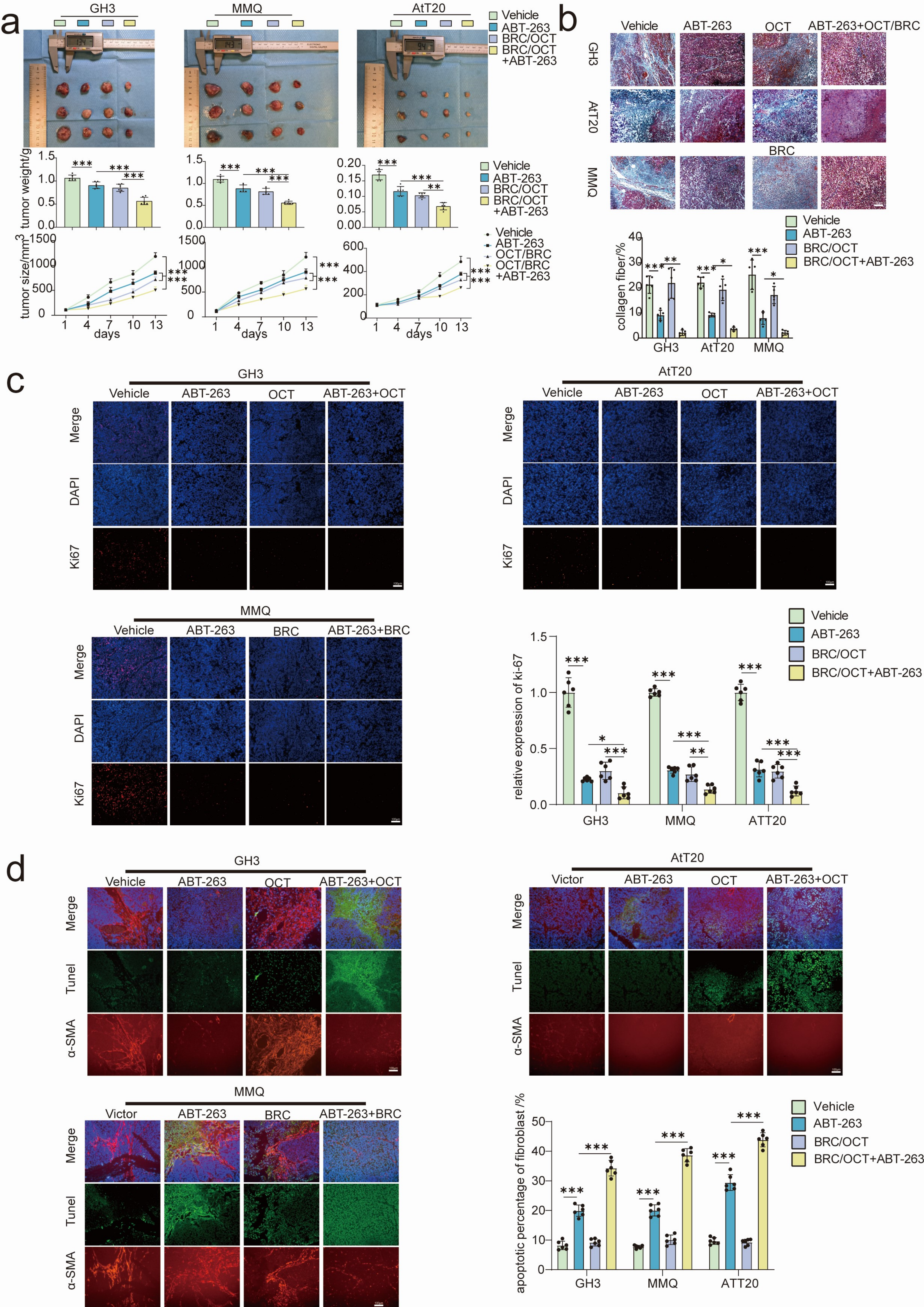

a
GH3
MMQ
AtT20
Vehicle
ABT-263
BRC/OCT
BRC/OCT +ABT-263
tumor weight/g
tumor size/mm3
days
OCT/BRC
OCT/BRC +ABT-263
b
Vehicle
ABT-263
OCT
ABT-263+OCT/BRC
BRC
collagen fiber/%
BRC/OCT+ABT-263
c
ABT-263+OCT
ABT-263+BRC
Merge
DAPI
Ki67
relative expression of ki-67
ATT20
d
Victor
Tunel
α-SMA
apoptotic percentage of fibroblast /%

Figure S8. ABT-263 improved efficiency of clinical drugs for PA.

a Analysis of tumor size and weight after the combination of ABT-263 + clinical medication in nude mice with implants PA cells (n = 6). b Masson staining to detect PA collagen expression levels after the combination of ABT-263 + clinical medication (n = 6) (scale bar = 100μm). c Ki67 staining to detect PA cell activity after combination of ABT-263 + clinical medication (n = 6) (scale bar = 100μm). d α-SMA (red) and tunel (green) co-staining to detect the extent of fibroblast apoptosis, blue for DAPI. Data were expressed as mean ± SD. One-way ANOVA was used for multi-sample comparison and Student's t-test was used for comparison between two groups. Statistical significance was considered to be indicated by a value of $p < 0.05$. * P<0.05, ** P<0.01, *** P<0.005.BRC: Bromocriptine, OCT: Octreotide.
