# Supplementary figures and images for: Tumor-Associated Fibroblast-Derived Exosomal circDennd1b Promotes Pituitary Adenoma Progression by Modulating the miR-145-5p/ONECUT2 Axis and Activating the MAPK Pathway
Source: Cancers (Basel). 2023 Jun 27;15(13):3375. doi: 10.3390/cancers15133375 (PMC10340501; doi:10.3390/cancers15133375)

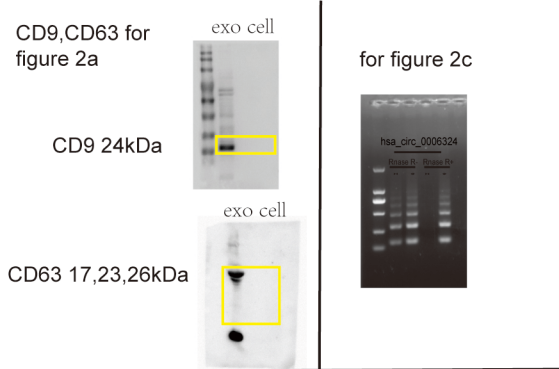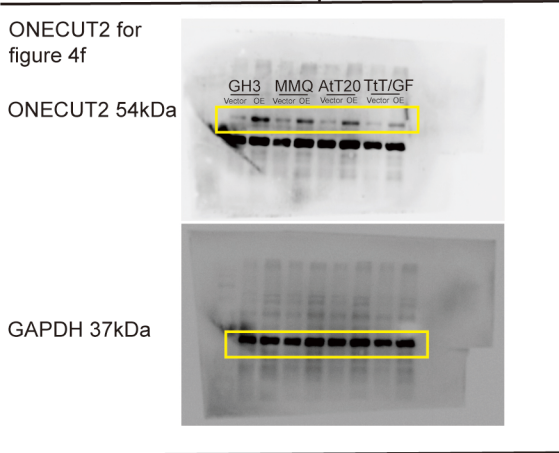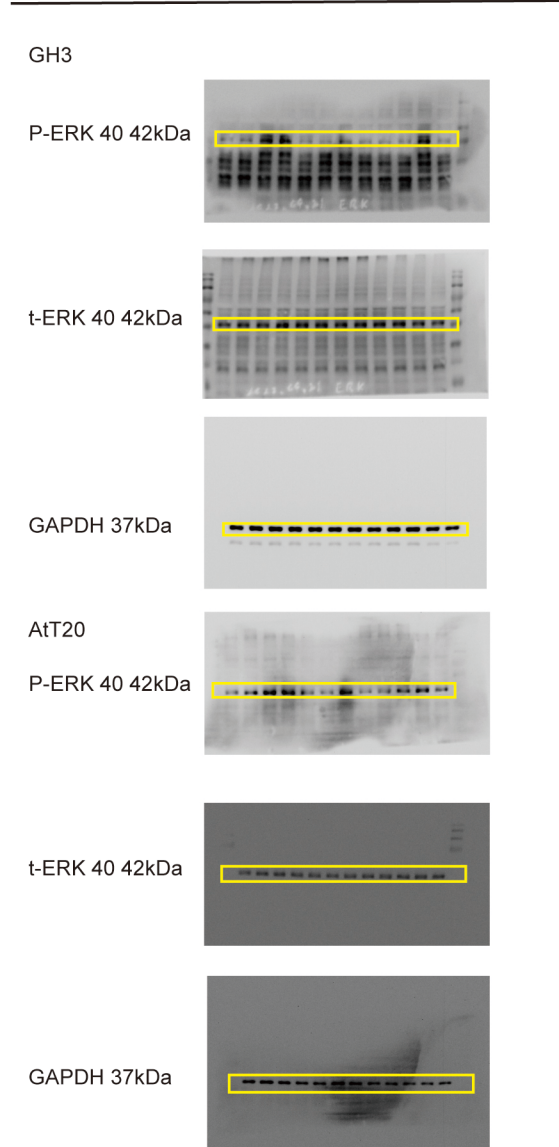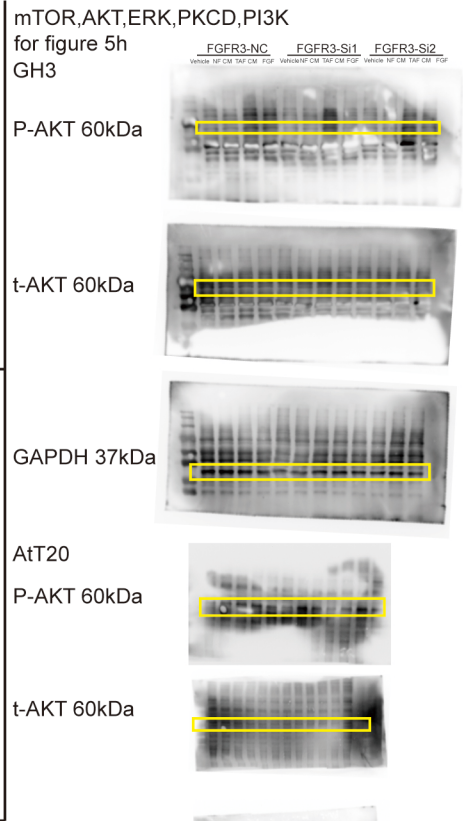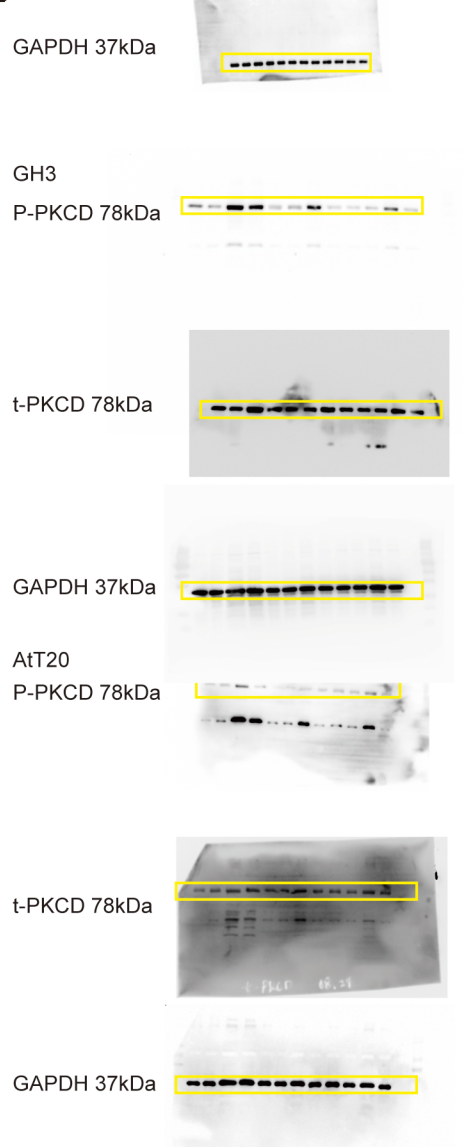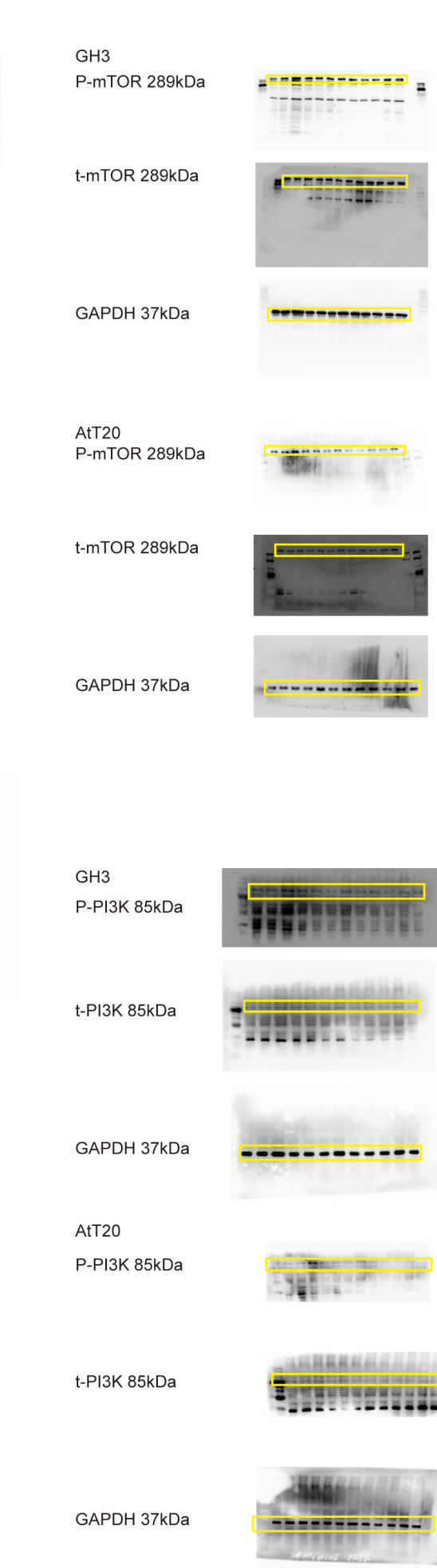

Supplement: Supplementary file 1 [file cancers-15-03375-s001.zip › Western Blot and gel.pdf]
